# The efficacy of trastuzumab deruxtecan in Chinese breast cancer patients: a real-world multicenter study

**DOI:** 10.3389/fonc.2025.1582498

**Published:** 2025-08-18

**Authors:** Miao He, Xiaohui Liu, Yan Zhang, Zhenyu Shao, Tiantian Sun, Yu Hu

**Affiliations:** ^1^ Department of Medical Oncology, Qilu Hospital, Cheeloo College of Medicine, Shandong University, Jinan, Shandong, China; ^2^ Department of Medical Oncology, Qilu Hospital (Qingdao), Cheeloo College of Medicine, Shandong University, Qingdao, China; ^3^ Breast Center, Central Hospital Affiliated to Shandong First Medical University, Jinan, China; ^4^ Department of Radiotherapy, Qilu Hospital, Cheeloo College of Medicine, Shandong University, Jinan, Shandong, China; ^5^ Department of Chemotherapy, Zibo Central Hospital, Zibo, Shandong, China

**Keywords:** trastuzumab deruxtecan, antibody-drug conjugate, DCR, ILD, breast cancer

## Abstract

**Introduction:**

In mainland China, trastuzumab deruxtecan was first authorized in February 2023 as a monotherapy for the treatment of patients with HER-2-positive adult breast cancer who were either unresectable or had metastasized after receiving one or more anti-HER-2 treatments. In July 2023, trastuzumab deruxtecan was approved for the treatment of patients with metastatic disease who had undergone at least one previous systemic therapy, as well as those with unresectable or metastatic adult breast cancer who had low expression of HER-2 and who had experienced a relapse within six months of finishing adjuvant chemotherapy.

**Methods:**

The study included seven participants with HER-2 low expression breast cancer and eighteen participants with HER-2-positive advanced breast cancer from six study centers in Shandong Province, China. Efficacy and safety data on trastuzumab deruxtecan were also gathered. The study involved intravenous injection of trastuzumab deruxtecan at a dosage of 5.4 mg/kg every three weeks until the disease progressed or the drug's toxicity became unmanageable, whichever came first.

**Results and Discussion:**

During a 8-month follow-up period, the disease control rate for patients with HER-positive breast cancer was 88.89% (16/18). The disease control rate for patients with HER-2 low-expressing breast cancer was 85.71% (6/7). The most common adverse reactions were gastrointestinal reactions, such as nausea, which occurred in 64.00% (16/25), and interstitial lung disease, which had a probability of occurring in 4.00% (1/25). In this real-world study, trastuzumab deruxtecan showed favorable efficacy and safety in both HER-2-positive breast cancer and HER-2 low-expressing breast cancer.

## 1 Introduction

HER-2, a receptor found in 25-30% of breast cancer cases, is often associated with poorer clinical outcomes (1, 2). Despite the advent of targeted therapies like trastuzumab (3), lapatinib (4), patuzumab (5) and Trastuzumab Emtansine(T-DM1) (6), many patients continue to experience disease progression, highlighting the urgent need for more effective treatments.

Trastuzumab Deruxtecan(T-DXd) is a novel antibody-drug conjugate (ADC) that pairs trastuzumab, a monoclonal antibody targeting HER-2, with deruxtecan, a potent agent that inhibits topoisomerase I (7). By binding to HER-2 receptors on tumor cells, T-DXd delivers the cytotoxic drug directly to the cancer cells, and it also exerts a ‘ bystander effect ‘, targeting surrounding HER-2-positive cells (8).

T-DXd received approval in mainland China in early 2023 to treat HER-2-positive breast cancer, especially in patients whose disease is metastatic or incurable. The treatment was initially offered to patients who had previously received anti-HER-2 treatments. By mid-2023, however, the indication was broadened to encompass patients with HER-2-low expression (IHC 1+ or IHC 2+/ISH-), including those who experienced a relapse within six months of adjuvant chemotherapy, and those who had received at least one round of systemic therapy during the metastatic phase. This wider approval represents a major advancement in the treatment of breast cancer by providing patients with few options with additional therapeutic options.As of right now, T-DXd is approved globally to treat a number of malignancies, including lung, stomach, breast, and gastroesophageal junction adenocarcinoma (9–13).

T-DXd has proven to be highly effective in several important clinical trials. The 2019 San Antonio Conference revealed that, despite participants having had an average of six previous lines of therapy, T-DXd achieved an objective response rate (ORR) of 60.9%, a disease control rate (DCR) of 97.3%, and a progression-free survival (PFS) of 16.4% (14). In 2020, follow-up data revealed even better results: overall survival (OS) was 24.6 months, PFS was 19.4 months, and ORR was 61.4%. Nonetheless, drug-related pneumonia or interstitial lung disease (ILD) affected 15.8% of patients, highlighting the necessity of respiratory health monitoring during treatment (15).

The DESTINY-Breast03 trial further validated T-DXd’s superiority over T-DM1 in patients with advanced HER-2-positive breast cancer who had previously received treatment with trastuzumab and taxanes. T-DXd showed a median PFS evaluated by the researchers was 29.0 months, while it was 7.2 months for the T-DM1 group. The median OS was 52.6 months and 42.7 months respectively (16, 17). These results played a pivotal role in T-DXd’s FDA approval for patients with prior anti-HER-2 treatments. Thrombocytopenia, anemia, and neutropenia were the most often reported serious adverse effects.

In patients who had previously had therapy with T-DM1, T-DXd outperformed the physician’s selected treatment (TPC) in the DESTINY-Breast02 trial. The median progression-free survival (PFS) for T-DXd was 16.7 months, which was much longer than the 5.5 months for TPC, and the ORR was 74.1%, as opposed to 27.2% for TPC. This trial further established T-DXd as the recommended treatment for individuals who had not responded to previous treatments such as T-DM1; the median overall survival (OS) was 35.7 months as opposed to 25.0 months for TPC (18, 19).

The approval of T-DXd for patients with low HER-2 expression (IHC 1+ or IHC 2+/ISH-), a previously underserved category, represents a revolutionary step. Low HER-2 expression is seen in 45–55% of breast cancer cases in China, highlighting a serious therapeutic gap. T-DXd was shown to be successful for these patients in the DESTINY-Breast04 study, with a median OS of 23.4 months versus 16.8 months and a median PFS of 9.9 months compared to 5.1 months with physician-choice therapies. These findings highlight T-DXd’s revolutionary impact in changing the therapeutic landscape for patients with low HER-2 malignancies and have increased treatment options for these individuals (20). Among Asian patients with hormone receptor-positive mBC, the median PFS of T-DXd and TPC were 10.9 and 5.3 months, respectively, and 10.9 and 4.6 months, respectively in the entire Asian population. Among these two populations, the median OS of patients with T-DXd was not achieved, and the median OS of patients with TPC was 19.9 months (21).In the recent DESTINY-Breast06study when comparing T-DXd with TPC(capecitabine/paclitaxel), the median PFS of T-DXd was significantly prolonged in patients with extremely low HER-2 expression (IHC 0+) (13.3 months vs 8.5 months). Support the FDA’s approval of T-DXd for the indication of breast cancer with ultra-low expression of HR+/HER-2- in January 2025 (22).

With an emphasis on patients with HER-2-positive and HER-2-low expressing breast cancer, this study attempts to further assess T-DXd’s safety and effectiveness in the Chinese population, building on the increasing body of evidence. T-DXd is in a strong position to meet the unmet requirements of patients with metastatic or incurable disease because of its growing indications and growing clinical data. This study aims to advance global knowledge of T-DXd’s potential in both HER-2-positive and HER-2-low subgroups by providing additional insights into its practical utilization.

## 2 Materials and methods

### 2.1 Study population

This observational study took place between November 2023 and October 2024 at eight medical institutes in Shandong Province, China. The study was approved by the ethical committee of Qilu Hospital, Shandong University. All participants provided informed consent after being fully apprised of the study’s purpose and procedures. The trial followed Good Clinical Practice (GCP) principles throughout.

The trial included adult patients with metastatic or inoperable breast cancer. Participants have to have HER-2 IHC 3+ or HER-2 IHC 2+ and a confirmed positive fluorescence *in situ* hybridization (FISH) result. Furthermore, patients with low HER-2 expression (IHC 1+ or IHC 2+/ISH-) who had relapsed within six months of adjuvant chemotherapy or had undergone at least one course of systemic therapy during the metastatic phase were eligible.

Eligibility criteria for participation included being 18 years or older, with an Eastern Cooperative Oncology Group (ECOG) performance status of 0 or 1. Each participant had to have at least one measurable lesion according to the RECIST 1.1 criteria.

Patients needed to meet the following clinical parameters: left ventricular ejection fraction (LVEF) ≥50%, absolute neutrophil count ≥1500 cells/mm³, hemoglobin levels ≥90 g/L, platelet count ≥100,000 cells/mm³, and liver enzyme levels (AST and ALT) ≤2.5 times the upper limit of normal (ULN). Bilirubin levels were required to be ≤1.5×ULN.

Within three weeks of screening, patients could not have received anti-HER-2 antibody-drug conjugates (ADCs), chemotherapy, hormonal therapy, radiation, or surgery. Patients with symptomatic chronic heart failure, severe arrhythmias, substantial systemic illnesses, human immunodeficiency virus, hepatitis B infection, pregnancy, breastfeeding, known hypersensitivity to T-DXd, or other contraindications were excluded.

### 2.2 Treatment methods

During the first cycle, patients received an intravenous infusion of T-DXd at a rate of 5.4 mg/kg over 90 minutes. In following cycles, the infusion period was shortened to 30 minutes. Treatment was scheduled every three weeks until disease progression or severe adverse events developed, whichever came first. Patients who encountered severe adverse events that required treatment termination were classed as having uncontrolled toxicity.

To avoid or reduce infusion-related responses, premedication for each infusion consisted of a mix of analgesics, antipyretics, and antihistamines. According to the study design, palliative care, ondansetron, and palonosetron were among the additional supportive medications that were given as needed.

### 2.3 Efficacy and toxicity

Evaluations were carried out utilizing the RECIST 1.1 criteria in order to gauge the tumor response (23). Twelve-week intervals were allotted for these exams, with more frequent evaluations occurring if there was indication of accelerated illness progression.

The disease control rate (DCR) and objective response rate (ORR) were the main indicators of efficacy in this study. The DCR represented the percentage of patients with an evaluable response who obtained either a complete response (CR), partial response (PR), or stable disease (SD). The percentage of CR and PR is reflected in the ORR. Complete Response (CR): This is defined as the total disappearance of all target lesions, no appearance of new lesions, and stable tumor markers for a minimum duration of 4 weeks.Partial Response (PR): A decrease of at least 30% in the largest diameter of the target lesions, sustained for a minimum of 4 weeks. Stable Disease (SD): A condition where the sum of the diameters of target lesions changes by no more than a 30% decrease or a 20% increase.

Furthermore, adverse events (AEs) were closely tracked and classified using the National Cancer Institute’s Common Terminology Criteria for Adverse Events (CTCAE), version 5.0. A tiny percentage of patients on T-DXd may experience interstitial lung disease. We performed a baseline assessment before to treatment, which comprised a baseline imaging examination, a baseline pulmonary function test, a baseline laboratory test, and a collection of the respiratory system history. Frequent imaging monitoring was performed once every 6–8 weeks during the course of treatment. It is imperative that lung function tests be performed again very away if there are any new respiratory symptoms or if imaging changes are suspected.

### 2.4 Statistical analysis

For the analysis of efficacy outcomes, statistical comparisons were made between the HER-2-positive and HER-2-low expressing breast cancer subgroups. Categorical data were analyzed using either Chi-square tests or Fisher’s exact tests, depending on the characteristics of the data. The Kaplan-Meier test was used to estimate PFS. The statistical and graphical software used was SPSS software (version 24.0), Graphpad, and the statistical significance threshold was set at p≤ 0.05.

## 3 Results

### 3.1 Attributes of patients

A total of 25 participants were included in the study, comprising 18 individuals with HER-2-positive breast cancer and 7 with HER-2-low expressing breast cancer. The participants’ ages ranged from 33 to 73 years, with a median age of 55. Among the group, 60% (15/25) were hormone receptor-positive. Every subject had distant metastases; 40% (10/25) had brain involvement, and 92% (23/25) had visceral metastases. Before enrolling in the study, every patient had received at least one prior line of systemic treatment. **Table 1** lists all of the individuals’ baseline characteristics in detail.

**Table 1:** Baseline clinicopathological and disease characteristics of 25 breast cancer patients enrolled in the study.

Characteristics	Total (N/%)
Median age(range), years	55(33-73)
21–45 years	5(20.00%)
46–60 years	12(48.00%)
>60 years	8(32.00%)
ECOG performance status	
0-1	25(100%)
≥2	0(0%)
Hormone-receptor status	
-	10(40.00%)
+	15(60.00%)
Lines of T-DXd treatment in mBC	
1	0(0%)
2	2(8.00%)
≥3	23(92.00%)
Distant metastasis	
No	0(0%)
Yes	25(69.31%)
Visceral metastasis	
No	2(8.00%)
Yes	23 (92.00%)
Brain metastasis	
Yes	10(40.00%)
No	15(60.00%)

ECOG, Eastern Cooperative Oncology Group; mBC, metastatic breast cancer.

### 3.2 Treatment administration

Participants have received a variety of treatment plans prior to enrollment. **Table 2** provides an overview of their past medical history and treatment measures. The median follow-up time was 9.1 months.

**Table 2:** Previous treatment for all patients.

Previous treatment		Low expression of HER-2	Proportion (%)	High expression of HER-2	Proportion (%)
		(N=7)		(N=18)	
Endocrine therapy		7	100.00	7	38.89
Previous use of chemotherapeutic drugs	Paclitaxel	6	85.71	17	94.44
	Anthracycline	7	100.00	8	44.44
	Cyclophosphamide	6	85.71	8	44.44
	Pyrimidines	7	100.00	12	66.67
	Vinorelbine	1	14.29	4	22.22
	Platinum	4	57.14	9	50.00
Previous use of Targeted therapy	Trastuzumab	0	0	18	100.00
	Pertuzumab	0	0	14	77.78
	Pyrotinib	0	0	12	66.67
	Lapatinib	0	0	2	11.11
CDK4/6 inhibitor		6	85.71	3	16.67
Antiangiogenic		3	42.86	2	11.11
ADC		0	0	10	55.56

### 3.3 Clinical efficacy

**Figure 1:**
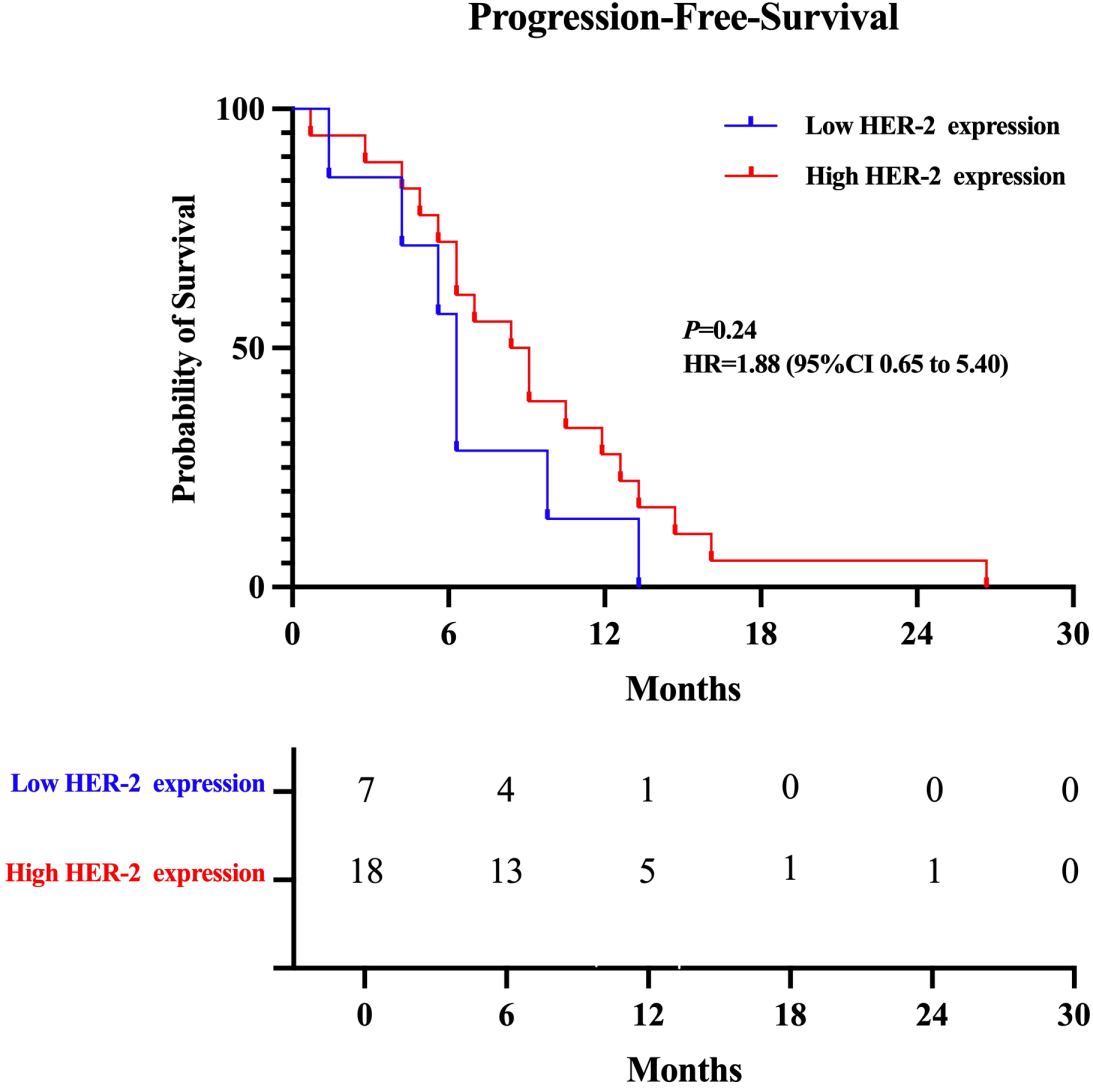
Kaplan-Meier plot of the progression-free survival (PFS) of T-Dxd in patients with low and high HER-2 expression.

Key outcomes such ORR, DCR, and the median progression-free survival (mPFS) for both HER-2-positive and HER-2-low expressing subsets of breast cancer were used to evaluate the therapeutic efficacy of T-DXd. The mPFS was 6.3 months for patients with low HER-2 expression and 8.7 months for the high-expression group. As detailed in **Table 3** and **Figure 1**. There was no statistically significant difference in ORR (p=0.899 95%CI 0.19 ~ 4.38) DCR (p=0.270 95%CI 0.22 ~ 1.53) and mPFS (p= 0.24 95%CI 0.65 -5.40) compared to patients with high and low HER-2 expression. There were also no statistically significant differences observed between subgroups when comparing their clinical outcomes.

**Table 3:** Evaluation of efficacy in patients with different clinicopathologic and disease characteristics.

Characteristics	Low expression of HER-2(N=7) (N/%)	ORR	P	DCR	P	mPFS(days)	P	High expression of HER-2 (N=18) (N/%)	ORR	P	DCR	P	mPFS (days)	P
Sex														
Male	1 (14.29%)	0%	–	100.00%	0.868	42	0.286	0 (0%)	0%	–	0%	–	–	–
Female	6 (85.71%)	33.33%		83.33%		189		18 (100%)	44.44%		88.89%		262.5	
ECOG performance status														
0-1	7 (100%)	28,57%	–	85.71%	–	189		18 (100%)	44.44%	–	88.89%	–	262.5	–
≥2	0 (0%)	0%		0%		–		0 (0%)	0%		0%		–	
Tumor histology														
Ductal	6 (85.71%)	33.33%	–	83.33%	0.868	189	0.571	17 (94.44%)	47.06%	–	88.24%	0.998	273	0.44
Other	1 (14.29%)	0%		100.00%		126		1 (5.56%)	0%		100.0%		147	
Hormone receptor status														
–	0 (0%)	0%	–	0%	–	–	–	10 (55.56%)	40.00%	0.218	90.00%	0.312	231	0.696
+	7 (100.00%)	28,57%		85.71%		189		8 (44.44%)	50.00%		87.50%		262.5	
Ki-67														
<14	1 (14.29%)	0%	–	100.00%	0.868	294	0.571	0 (0%)	0%	–	0%	–	–	–
≥14	6 (85.71%)	33.33%		83.33%		178.5		18 (100%)	44.44%		88.89%		262.5	
Histopathological grade														
≤2	4 (57.14%)	25%	>0.99	75.00%	0.725	189	1.00	8 (44.44%)	50.00%	0.218	90.00%	0.312	325.5	0.274
>2	3 (42.86%)	33.33%	9	100.00%		168		10 (55.56%)	40.00%		87.50%		231	
Visceral Metastasis														
No	1 (14.29%)	0%	–	100.00%	0.868	126	0.57	1 (5.56%)	0%	–	100.00%	0.998	273	0.889
Yes	6 (85.71%)	33.33%		83.33%		189		17 (94.44%)	47.06%		88.24%		252	
Number of Visceral Metastasis														
1	3 (42.86%)	0%	–	66.67%	0.657	189	0.70	7 (38.89%)	28.57%	0.218	85.71%	0.182	315	1.0
≥2	3 (42.86%)	66.67%		100.00%		168		10 (55.56%)	60.00%		90.00%		199.5	
Lines of T-DXd treatment in MBC														
1-2	2 (28.57%)	0%	–	100.00%	0.797	294	0.190	0 (0%)	0%	–	0%	–	–	–
≥3	5 (71.43%)	40.00%		80.00%		168		18 (100.00%)	44.44%		88.89%		262.5	
Brain metastasis														
Yes	1	0%	–	100.00%	0.999	168	0.857	9	33.33%	0.982	77.78%	0.610	273	0.605
NO	6	33.33%	–	83.33%	0.999	189	0.857	9	55.56%	0.982	88,.89%	0.610	252	0.605

ECOG, Eastern Cooperative Oncology Group; mBC, metastatic breast cancer.

### 3.4 Safety

A total of 25 patients were monitored for treatment-related adverse events (AEs). In total, 61.1% (11/18) of the HER-2-positive group and 71.43% (5/7) of the HER-2-low expressing group experienced treatment-related adverse events. Gastrointestinal side effects were the most frequently reported. 71.43% (5/7) of the patients with low HER-2 expression and 61.1% (11/18) of the patients with HER-2-positive expressed nausea. Vomiting occurred in 44.44% (8/18) of HER-2-positive patients and 42.86% (3/7) of the HER-2-low expressing patients. 28.57% (2/7) of patients with HER-2-low expression and 27.78% (5/18) of patients with HER-2-positive expressed fatigue. However, all of these reactions were grade 1-2.

Other adverse events included anemia in 24% (6/25) of the total cohort, and neutropenia in 20% (5/25) of patients. Other adverse reactions included anemia in 24% (6/25) of patients and neutropenia in 20% (5/25), with one case rated grade 3. Hematologic and gastrointestinal toxicities were most common, but most were grade 1-2. Of note, one (1/25) patient experienced dyspnea, which resolved completely after two days of oxygen therapy and steroid medication. This adverse reaction was rated as grade 3.

A full list of the adverse events is provided in **Table 4**. Overall, the adverse events were manageable, and symptomatic treatments such as Aprepitant and ondansetron were used to alleviate discomfort, enabling most patients to continue their treatment with T-DXd.

**Table 4:** Trastuzumab Deruxtecan-related adverse events of all grades.

Grading of adverse events	HER-2 low expression(N=7) (N/%)	HER-2 positve (N=18) (N/%)
Leukopenia		
–	5 (71.43%)	15 (83.33%)
1-2	2 (28.57%)	2 (11.11%)
3-4	0 (0%)	1 (5.56%)
Neutropenia		
–	5 (71.43%)	15 (83.33%)
1-2	2 (28.57%)	2 (11.11%)
3-4	0 (0%)	1 (5.56%)
Anemia		
–	5 (71.43%)	14 (77.78%)
1-2	2 (28.57%)	4 (22.22%)
3-4	0 (0%)	0 (0%)
Thrombocytopenia		
**-**	7 (100.00%)	16 (88.89%)
1-2	0 (0%)	2 (11.11%)
3-4	0 (0%)	0 (0%)
Abnormal liver function		
–	7 (100.00%)	15 (83.33%)
+	0 (0%)	3 (16.67%)
Diarrhea		
–	7 (100.00%)	18 (100.00%)
+	0 (0%)	0 (0%)
Nausea		
–	2 (28.57%)	7 (38.89%)
+	5 (71.43%)	11 (61.11%)
Vomit		
–	4 (57.14%)	10 (55.56%)
+	3 (42.86%)	8 (44.44%)
Fatigue		
–	5 (71.43%)	13 (72.22%)
+	2 (28.57%)	5 (27.78%)
Dyspnea		
–	7 (100.00%)	17 (94.44%)
+	0 (0%)	1 (5.56%)
LVEF decreases		
–	7 (100.00%)	18 (100%)
+	0 (0.00%)	0 (0%)

LVEF, left ventricular ejection fraction.

## 4 Discussion

T-DXd has emerged as a significant therapeutic option for metastatic breast cancer overexpressing HER-2, demonstrating robust clinical efficacy in recent studies. Typically, initial treatment for HER-2-positive breast cancer consists of a combination of monoclonal antibodies targeting HER-2—pertuzumab and trastuzumab—alongside chemotherapy based on taxanes (5, 24, 25).

However, as shown by the results of the EMILIA trial, T-DM1 is frequently regarded as the next line of treatment for patients whose condition worsens following first-line medications (6).

Recent studies have highlighted the better PFS reported with T-DXd. In particular, 75.8% of patients treated with T-DXd were progression-free and alive at 12 months, compared to only 34.1% in the T-DM1 cohort. Furthermore, an astonishing 96.6% of patients who received T-DXd had disease control. These findings were consistent across all subgroups, including patients with varying hormone receptor statuses, those who had previously received pertuzumab, and those with visceral or brain metastases (26).

T-DXd showed good efficacy in both HER-2-positive and HER-2 low expression metastatic BC patients (16, 20). Our work supports these findings by demonstrating encouraging results in both HER-2-positive and HER-2-low expressing breast cancer patients.

One significant feature of T-DXd is its ability to efficiently target tumors with low levels of HER-2. The DESTINY-Breast04 trial reported that the median PFS of patients with low HER-2 expression was 9.9 months and the DCR was 87.9%. In our study, while the DCR for the HER-2-low subgroup was slightly lower at 85.71% and the median PFS was 6.3 months, these results still highlight the potential of T-DXd for this previously underrepresented group of patients (20). Several factors could explain the minor discrepancies found in our investigation. Specifically, the HER-2-low subgroup was tiny, with only seven patients. Furthermore, a large proportion of patients got T-DXd as a third-line treatment or later, which could have impacted the clinical results seen.

Brain metastases continue to be a significant difficulty in treating metastatic HER-2-positive breast cancer. Animal studies have shown that T-DXd can slow tumor progression and enhance survival outcomes in both HER-2-positive and HER-2-negative brain metastases (27), We found no statistically significant difference in PFS between patients with and without brain metastases in our cohort. This lack of significance is most likely owing to the small sample size, which reduced the statistical power of our analysis. Nonetheless, our findings support the notion that T-DXd could be a viable treatment option for individuals with brain metastases, though bigger trials are required to confirm these promising findings.

In terms of safety, the adverse event profile of T-DXd corresponds with that of T-DM1, albeit the frequency of major side effects such as interstitial lung disease (ILD) or pneumonia was significantly greater in some studies, including 10.5% of patients in the T-DXd group against 1.9% for T-DM1 (26). In the Chinese population, common gastrointestinal issues like nausea, vomiting, and fatigue were reported. In the HER-2-low category, 52.6% had grade 3 or higher adverse events, while 12.1% developed ILD or pneumonia (20) Our findings revealed a lower occurrence of grade 3 or higher adverse events, with the majority of side effects being moderate (grades 1 or 2). This could be linked to the proactive use of antiemetic medications to alleviate the intensity of gastrointestinal symptoms.

While the adverse effects were usually treatable, problems such as ILD remain significant concerns. Symptoms such as shortness of breath, cough, and fatigue can worsen in severe cases, sometimes leading to respiratory distress (28, 29) The precise processes behind these toxicities remain unknown, and more research is needed to better understand the dangers associated with these side effects (30, 31).

The median time to first ILD/pneumonia was 5.5 months. Most of the T-DXd-related ILD/pneumonia adverse effects were of modest grade (32). Several studies have reported factors that may be associated with T-DXd-associated ILD/pneumonia, including T-DXd dose, baseline oxygen saturation (SpO2), moderate/severe renal impairment, and the presence of certain pulmonary comorbidities (32, 33). Only one patient developed dyspnea in this study, which may be due to the small number of cases and the fact that follow-up did not reach the median time for ILD/pneumonia.

This retrospective study has some limitations. Firstly, since T-DXd is not included in China’s medical insurance, the actual number of patients using it is relatively small, which may lead to result deviations. In addition, the follow-up time of the patients was relatively short. Therefore, the long-term efficacy of T-DXd in different types of populations, such as PFS or OS, requires further verification through large prospective cohort studies.

This real-world study provides solid evidence for T-DXd’s therapeutic benefits in treating metastatic breast cancer, both HER-2-positive and HER-2-low expressing subtypes. While the small number of patients and short duration of follow-up are acknowledged limitations, the findings indicate that T-DXd is a potential therapeutic option for people with metastatic breast cancer. Additional large-scale and long-term investigations are required to validate and expand on these favorable findings.

## Data Availability

The raw data supporting the conclusions of this article will be made available by the authors, without undue reservation.
